# Human Microbe-Disease Association Prediction With Graph Regularized Non-Negative Matrix Factorization

**DOI:** 10.3389/fmicb.2018.02560

**Published:** 2018-11-01

**Authors:** Bin-Sheng He, Li-Hong Peng, Zejun Li

**Affiliations:** ^1^The First Affiliated Hospital, Changsha Medical University, Changsha, China; ^2^School of Information Engineering, Changsha Medical University, Changsha, China; ^3^College of Information Science and Engineering, Hunan University, Changsha, China; ^4^School of Computer and Information Science, Hunan Institute of Technology, Hengyang, China

**Keywords:** microbe, disease, association prediction, graph regularization, matrix factorization

## Abstract

A microbe is a microscopic organism which may exists in its single-celled form or in a colony of cells. In recent years, accumulating researchers have been engaged in the field of uncovering microbe-disease associations since microbes are found to be closely related to the prevention, diagnosis, and treatment of many complex human diseases. As an effective supplement to the traditional experiment, more and more computational models based on various algorithms have been proposed for microbe-disease association prediction to improve efficiency and cost savings. In this work, we developed a novel predictive model of Graph Regularized Non-negative Matrix Factorization for Human Microbe-Disease Association prediction (GRNMFHMDA). Initially, microbe similarity and disease similarity were constructed on the basis of the symptom-based disease similarity and Gaussian interaction profile kernel similarity for microbes and diseases. Subsequently, it is worth noting that we utilized a preprocessing step in which unknown microbe-disease pairs were assigned associated likelihood scores to avoid the possible negative impact on the prediction performance. Finally, we implemented a graph regularized non-negative matrix factorization framework to identify potential associations for all diseases simultaneously. To assess the performance of our model, cross validations including global leave-one-out cross validation (LOOCV) and local LOOCV were implemented. The AUCs of 0.8715 (global LOOCV) and 0.7898 (local LOOCV) proved the reliable performance of our computational model. In addition, we carried out two types of case studies on three different human diseases to further analyze the prediction performance of GRNMFHMDA, in which most of the top 10 predicted disease-related microbes were verified by database HMDAD or experimental literatures.

## Introduction

Antonie Van Leeuwenhoek, the father of microbiology, was the first to discover, observe, describe, study, and conduct scientific experiments with microbes, using simple single-lensed microscopes of his own design in 1673 (Leeuwenhoek, 1683-1775). From then on, with the development of biological theory and technology, a great mass of microbes has been discovered. It has been suggested that the amount of organisms living below the Earth's surface is comparable with the amount of life on or above the surface (Gold, 1992). As we know, microbes are very closely related to humans in many fields, such as food production (Smid and Lacroix, 2013), water treatment (Tabatabaei et al., 2010), energy (Tanaka, 1999), and human health (Thiele et al., 2013). Especially, many studies have demonstrated that one of the most important effects of microbes on humans is the associations between microbes and complex human diseases. For example, Boleij et al. (2015) proved that the *Bacteroides fragilis* toxin gene is associated with colorectal neoplasia, especially in late-stage colorectal cancer (CRC). Moreover, Galiana et al. (2014) found that *Actinomyces* can be as an indicator in the evolution of chronic obstructive pulmonary disease (COPD) patients because their study confirmed a strong association between the presence or absence of *Actinomyces* and the severity of the clinical condition. Another example is that periodontal pathogens *Porphyromonas gingivalis* and *Fusobacterium nucleatum* stimulate tumorigenesis of oral squamous cell carcinoma (OSCC) via direct interaction with oral epithelial cells through Toll-like receptors which is beneficial to the development of corresponding prevention and treatment schemes (Binder Gallimidi et al., 2015). Thus, due to the fact that detecting potential microbiological markers could help to provide a better understanding of the pathogenesis of diseases and the role played by the microbiota in its severity, it is of great significance to explore the potential associations between microbes and diseases. However, since traditional experimental methods always suffer from the time constraints and capital limitations, proposing novel computational models is able to be an effective complement for uncovering potential microbe-disease associations. Recently, many feasible and effective prediction models have been developed by researchers.

In the last few years, some prediction models were proposed based on network analysis. Ma et al. (2017) developed an analysis method based on the microbe-based human disease network (Human Microbe Disease Network, HMDN) to infer the associations between microbes and disease genes, symptoms, chemical fragments, and drugs. In the method, they first utilized a large-scale text mining-based method to build the microbe-disease association network, on which the cosine similarity was calculated for each disease pair to construct the HMDN. Taking microbe-disease gene association prediction as an example, the potential related disease genes of a microbe in the HMDN can be finally obtained by finding the highly overlapped genes among the microbe-related diseases in the gene-based human disease network (Human Gene Disease Network, HGDN). Besides, in a similar way, this analysis method can also be used between HMDN and symptom-based human disease network (Human Symptoms Disease Network, HSDN), chemical fragment-based human disease network (Human Chemical Fragments Disease Network, HCDN), and drug-based human disease network (Human Drug Disease Network, HDDN) to infer the associations between microbes and disease symptoms, chemical fragments, and drugs, respectively. However, the prediction performance of this analysis method is limited by the small microbe-based disease network. Thereafter, Chen et al. (2017a) was the first to propose a computational model of KATZ measure for Human Microbe-Disease Association prediction (KATZHMDA) on a large scale. Firstly, they integrated the known microbe-disease associations network and Gaussian interaction profile kernel similarity networks of microbes and diseases into a heterogeneous graph. Through summarizing all walks with different weighted lengths (i.e., the walk with shorter length was assigned larger coefficient) for each microbe-disease pair, they finally calculated the association probability between each microbe and disease. Moreover, KATZHMDA is applicable for new diseases/microbes without known associations if there are additional available similarity information between the new disease/microbe and other diseases/microbes in the known microbe-disease association network. One limitation of KATZHMDA is that the optimal value of the number of walks is still hard to select. Later, Huang Z. A. et al. (2017) proposed a model of Path-Based Human Microbe-Disease Association Prediction (PBHMDA) by integrating known microbe-disease association network and Gaussian interaction profile kernel similarity network for microbes and diseases into a heterogeneous interlinked network in which a threshold was set to remove the edges that represent weak correlations. In the heterogeneous interlinked network, the weights of all paths between a microbe-disease pair were finally aggregated to represent the association probability between the microbe and the disease, while the weight of each path was calculated by multiplying the weights of all edges in the path without overlap and then penalizing the path with a decay coefficient. The limitation existing in PBHMDA is that it will cause bias to microbes or diseases with more known associations. Moreover, PBHMDA cannot work well for new microbes and new diseases.

In addition, some proposed models were not based on network analysis. Since the negative microbe-disease samples (i.e., microbe-disease pairs that are confirmed to have no associations) are unavailable, Wang et al. (2017) presented a semi-supervised learning-based computational model of Laplacian Regularized Least Squares for Human Microbe-Disease Association prediction (LRLSHMDA) by optimizing the Laplacian regularized least squares classifiers in microbe space and disease space. Finally, they used a simple weighted average operation on the above two optimal classifiers to obtain the final probability matrix that indicates the potential association probabilities between microbes and diseases. However, LRLSHMDA is still faced with the problem of being unable to be implemented to new diseases without known associated microbes. Similarly, with no need for negative samples, Huang Y. A. et al. (2017) developed the method of a Neighbor- and Graph-based combined Recommendation model for Human Microbe-Disease Association prediction (NGRHMDA) by combining two recommendation models that are neighbor-based collaborative filtering model and topological information-based model. In the neighbor-based collaborative filtering model, considering that different microbe-disease pairs may share the same microbes or diseases, they computed two association possibility matrices respectively from the microbe perspective and disease perspective and then averaged them to obtain a prediction matrix. While in the topological information-based model, they introduced a two-step diffusion approach on the microbe-disease bipartite graph to obtain another prediction matrix. Ultimately, the above two prediction matrices were simply averaged to get the final association possibilities for all microbe-disease pairs. What is worth noting is that NGRHMDA shares the same aforementioned disadvantage with LRLSHMDA.

In summary, all of the above models have their own limitations in predicting microbe-disease associations. Due to the lack of measurements for microbe/disease similarity, some models are only based on the Gaussian interaction profile kernel similarity of microbes and diseases that leads to unavoidable bias to those well-investigated diseases and microbes. Besides, some models cannot predict for new microbes/diseases and optimal parameters in some models are not easy to select. In this work, considering some of the above limitations, we developed a novel computational model of Graph Regularized Non-negative Matrix Factorization for Human Microbe-Disease Association prediction (GRNMFHMDA). First of all, the information of Gaussian interaction profile kernel similarity of microbes and diseases, symptom-based disease similarity and known microbe-disease associations in HMDAD (Ma et al., 2017) were combined as the input to start the whole prediction process. Here, after data preparation, the prediction process consists of two main steps, the preprocessing step and the step of GRNMF. In the preprocessing step, the weighted *K* nearest neighbor profiles for microbes and diseases were calculated to reconstruct the original adjacency matrix obtained based on the known microbe-disease associations so that we could avoid the possible negative impact on the final prediction performance from unknown microbe-disease pairs. While in the step of GRNMF, Tikhonov (*L*_2_) and graph Laplacian regularization were introduced into the standard NMF framework to obtain a smoother solution from matrix factorization and take full advantage of the geometric structure of our data, respectively. In addition, global leave-one-out cross validation (LOOCV), local LOOCV and two types of case studies were carried out to evaluate the prediction performance of our model. As a result, GRNMFHMDA obtained AUCs of 0.8715 (global LOOCV) and 0.7898 (local LOOCV). More than that, 9 (Asthma), 9 (Obesity), and 8 (Type 1 diabetes) out of the top 10 predicted disease-related microbes were confirmed by HMDAD or experimental literatures. Thus, it is obvious that our model would perform well in microbe-disease association prediction according to the aforementioned results.

## Materials and methods

### Method overview

**Figure 1:**
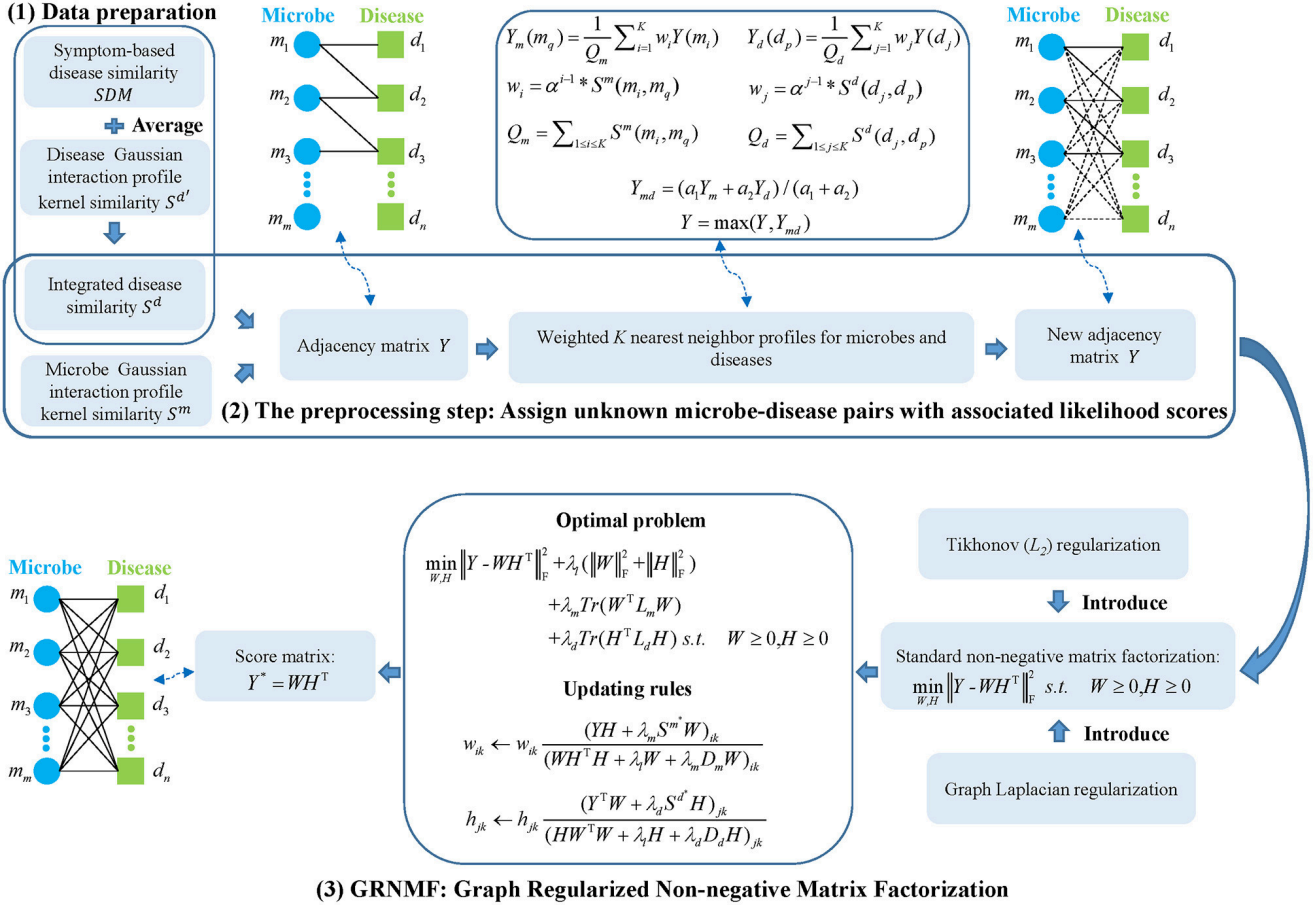
Flowchart of GRNMFHMDA model to predict the potential microbe-disease associations.

Here, to predict potential associations between microbes and diseases, the model of GRNMFHMDA (See Figure 1) can be decomposed into three steps: (1) data preparation, in which adjacency matrix, microbe similarity, and disease similarity were established; (2) the preprocessing step, in which unknown microbe-disease pairs were assigned with associated likelihood scores based on the calculation of weighted *K* nearest neighbor profiles for microbes and diseases; (3) GRNMF, in which Tikhonov (*L*_2_) and Graph Laplacian regularization were introduced into the standard NMF framework to obtain the final score matrix.

### Human microbe-disease associations

From the Human Microbe-Disease Association Database (HMDAD, http://www.cuilab.cn/hmdad) (Ma et al., 2017), we can download 483 known microbe-disease associations between 292 microbes and 39 human diseases. However, since some microbe-disease associations we downloaded are the same, there were only 450 known associations after removing the duplicate parts according to different evidences. In order to represent the associations information in a more convenient and efficient way, we defined an adjacency matrix *Y* ∈ *R*^m*n^, where *m* and *n* denoted the number of microbes and diseases, respectively. Moreover, the element *Y*(*m*_*i*_, *d*_*j*_) was set to 1 if microbe *m*_*i*_ and disease *d*_*j*_ had known association, otherwise 0.

### Gaussian interaction profile kernel similarity for microbes

There is a hypothesis that similar microbes (i.e., microbes exhibiting a similar pattern of interaction and non-interaction with the diseases of a microbe-disease association network) are inclined to be associated with the same disease, on which many previous studies had relied to construct the Gaussian interaction profile kernel similarity for microbes (Chen et al., 2017a; Huang Z. A. et al., 2017). In this article, based on the same assumption, we first represent the interaction profile for each microbe with a binary vector involving the association information between the microbe and each disease in the known microbe-disease association network. On the basis of the definition of adjacency matrix *Y*, the *i*th row vector (*Y*(*m*_*i*_) = (*Y*_*i*1_, *Y*_*i*2_, …, *Y*_*in*_)) can be used to denote the interaction profile of microbe *m*_*i*_. Thus, according to the method of van Laarhoven et al. (2011), the Gaussian interaction profile kernel similarity between microbe *m*_*i*_ and *m*_*j*_ can be defined as follows:

(1) $$\begin{array}{l} S^{m}\left(m_{i},m_{j}\right)=\exp\left(-\gamma_{m}{\left||Y\left(m_{i}\right)-Y\left(m_{j}\right)|\right|}^{2}\right) \end{array}$$

where

(2) $$\begin{array}{l} \gamma_{m}={\gamma \prime }_{m}/\left(\frac{1}{m}\overset{m}{\underset{i=1}{\sum }}{\left||Y\left(m_{i}\right)|\right|}^{2}\right) \end{array}$$

Here, γ_*m*_ is the adjustment coefficient that can be obtained by normalizing another bandwidth parameter γ′_*m*_.

### Gaussian interaction profile kernel similarity for diseases

The construction of the Gaussian interaction profile kernel similarity for diseases is based on the assumption that similar diseases (i.e., diseases exhibiting a similar pattern of interaction and non-interaction with the microbes of a microbe-disease association network) are more likely to be associated with similar microbes. Here, the interaction profile for each disease is also represented by a binary vector containing the association information between the disease and each microbe in the known microbe-disease association network. Based on the same method of van Laarhoven et al. (2011), the *j*th column vector (*Y*(*d*_*j*_) = (*Y*_1*j*_, *Y*_2*j*_, …, *Y*_*mj*_)) denotes the interaction profile of disease *d*_*j*_ and the Gaussian interaction profile kernel similarity between disease *d*_*i*_ and *d*_*j*_ can be defined as follows:

(3) $$\begin{array}{l} S^{d^{\prime }}\left(d_{i},d_{j}\right)=\exp\left(-\gamma_{d}{\left||Y\left(d_{i}\right)-Y\left(d_{j}\right)|\right|}^{2}\right) \end{array}$$

where

(4) $$\begin{array}{l} \gamma_{d}={\gamma \prime }_{d}/\left(\frac{1}{n}\overset{n}{\underset{j=1}{\sum }}{\left||Y\left(d_{j}\right)|\right|}^{2}\right) \end{array}$$

Similarly, γ_*d*_ is the adjustment coefficient that can be calculated by normalizing another bandwidth parameter γ′_*d*_.

### Integrated symptom-based disease similarity

As we have mentioned above, Gaussian interaction profile kernel similarity is used in our model to measure the similarity of microbes and diseases. However, since the Gaussian interaction profile kernel similarity is an association information-based measurement, it is essential to combine more types of microbe or disease similarities based on other available biological information. Indeed, according to different biological data, many researchers have developed their own method to measure the similarity of microbes or diseases. For instance, Zhou et al. (2014) proposed a model of symptom-based human disease network (HSDN) to measure the disease similarity based on co-occurrence of disease/symptom terms recorded in different literatures. In this work, we implemented HSDN to calculate symptom-based disease similarity (*SDM*) and then constructed a new disease similarity matrix (*S*^*d*^) by integrating *SDM* with S^*d*′^ in an average way according to the study of Chen et al. (2017a):

(5) $$\begin{array}{l} S^{d}=\frac{S^{d^{\prime }}+SDM}{2} \end{array}$$

### Weighted *K* nearest neighbor profiles for microbes and diseases

Due to the fact that values in interaction profiles of microbes or diseases without known associations are all zeros, the prediction performance may be affected to some extent. Considering that, to deal with the above mentioned problem, we came up with a preprocessing step to establish new interaction profiles both for microbes and diseases. For each microbe *m*_*q*_, we first find out its *K* nearest known microbes, each of which must has at least one known association. Next, the similarity information between *m*_*q*_ and its *K* nearest known microbes together with the information of their corresponding *K* interaction profiles are combined to calculate the new interaction profile as follows:

(6) $$\begin{array}{l} Y_{m}\left(m_{q}\right)=\frac{1}{Q_{m}}\sum_{i=1}^{K}w_{i}Y\left(m_{i}\right) \end{array}$$

where

(7) $$\begin{array}{l} w_{i}={\alpha^{i-1}}^{*}S^{m}\left(m_{i},m_{q}\right) \end{array}$$

(8) $$\begin{array}{l} Q_{m}=\sum_{1\leq i\leq K}S^{m}\left(m_{i},m_{q}\right) \end{array}$$

Here, *m*_1_ to *m*_*K*_ denote the *K* nearest known microbes of *m*_*q*_ which were sorted in descending order based on the similarity values between them. The function of the weight coefficient *w*_*i*_ is that the corresponding similarity value is assigned higher weight if *m*_*i*_ is more similar to *m*_*q*_. Besides, α is a decay term whose value is in the range of [0,1] and *Q*_*m*_ is the normalization term.

In a similar way, the new interaction profile for each disease *d*_*p*_ can be defined as follows:

(9) $$\begin{array}{l} Y_{d}\left(d_{p}\right)=\frac{1}{Q_{d}}\sum_{j=1}^{K}w_{j}Y\left(d_{j}\right) \end{array}$$

(10) $$\begin{array}{l} w_{j}={\alpha^{j-1}}^{*}S^{d}\left(d_{j},d_{p}\right) \end{array}$$

(11) $$\begin{array}{l} Q_{d}=\sum_{1\leq j\leq K}S^{d}\left(d_{j},d_{p}\right) \end{array}$$

After calculating the new interaction profiles from microbe perspective and disease perspective, we combine *Y*_*m*_ and *Y*_*d*_ as follows:

(12) $$\begin{array}{l} Y_{md}=\left(a_{1}Y_{m}+a_{2}Y_{d}\right)/\left(a_{1}+a_{2}\right) \end{array}$$

where *a*_1_ and *a*_2_ are two weight coefficient and both of them are set to 1 for simplicity.

Finally, to replace the element *Y*(*m*_*i*_, *d*_*j*_) = 0 with an associated likelihood score, we use the following equation to update the original adjacency matrix *Y*.

(13) $$\begin{array}{l} Y=\max\left(Y,Y_{md}\right) \end{array}$$

### GRNMF

As a common method, the purpose of the standard NMF is to find two non-negative matrices whose product is an optimal approximation to the original matrix (Sotiras et al., 2015; Xu et al., 2015). Therefore, the adjacency matrix *Y* ∈ *R*^m*n^ can be decomposed into two parts after implementing NMF, namely, *W* ∈ *R*^m*k^ and *H* ∈ *R*^n*k^ (*Y* ≈ *WH*^T^). Accordingly, we can further get the following standard optimization problem:

(14) $$\begin{array}{l} \underset{W,H}{\min}||Y-WH^{T}|{|}_{F}^{2}+L\left(W,H\right) \end{array}$$

where *L*(*W, H*) is a regularization term to prevent overfitting.

Here, motivated by the study of Xiao et al. (2017) and the standard NMF framework, we introduced other two terms, the Tikhonov (*L*_2_) (Guan et al., 2011) and graph Laplacian regularization (Cai et al., 2011), to predict microbe-disease associations. The utilizing of Tikhonov regularization aims to obtain a smooth solution (*W* and *H*), while the purpose of introducing graph regularization is to ensure a part-based representation through taking full advantage of the data geometric structure. Thus, we can construct the optimization problem of GRNMF as follows:

(15) $$\begin{array}{l} \underset{W,H}{\min}||Y-WH^{T}|{|}_{F}^{2}\text{+}\lambda_{l}\text{(}||W|{|}_{F}^{2}\text{+}||H|{|}_{F}^{2}\text{)}\\ \text{+}\lambda_{m}\overset{n}{\underset{i,p=1}{\sum }}{\left||w_{i}-w_{p}|\right|}^{2}{S^{m^{*}}}_{ip}\text{+}\lambda_{d}\overset{m}{\underset{j,q=1}{\sum }}{\left||h_{j}-h_{q}|\right|}^{2}{S^{d^{*}}}_{jq}\;s.t.\;W\geq \text{0,}H\geq \text{0} \end{array}$$

Here, λ_*l*_, λ_*m*_ and λ_*d*_ are the corresponding regularization coefficients. Besides,*w*_*i*_ and *h*_*j*_ are defined as *i*th rows of *W* and *j*th rows of *H*, respectively. In order to avoid negative affects to the prediction performance of our model, we introduced sparse weight matrices of *S*^d^^*^ and *S*^m^^*^that are constructed on the basis of the geometrical information of disease and microbe data spaces (*S*^*d*^ and *S*^*m*^), respectively. Then, Equation (14) can be transformed into:

(16) $$\begin{array}{l} \underset{W,H}{\min}||Y-WH^{T}|{|}_{F}^{2}\text{+}\lambda_{l}\text{(}||W|{|}_{F}^{2}\text{+}||H|{|}_{F}^{2}\text{)}\\ \text{+}\lambda_{m}Tr\text{(}W^{T}L_{m}W\text{)+}\lambda_{d}Tr\left(H^{\text{T}}L_{d}H\right)\;s.t.\;W\geq \text{0,}H\geq \text{0} \end{array}$$

where *Tr*(•) represents the trace of a matrix. Here, *L*_*m*_ and *L*_*d*_ are the corresponding graph Laplacian matrices for *S*^m^^*^ and *S*^d^^*^ that can be calculated as follows:

(17) $$\begin{array}{l} L_{m}=D_{m}-S^{m*} \end{array}$$

(18) $$\begin{array}{l} L_{d}=D_{d}-S^{d*} \end{array}$$

where *D*_*m*_ and *D*_*d*_ are the diagonal matrices whose entries are row (or column) sums of *S*^m^^*^ and *S*^d^^*^, respectively.

Based on the information of the nearest neighbor graph on a scatter of data points, researchers came up with a conclusion that local geometric structure is able to be effectively modeled (Cai et al., 2011; Li et al., 2017). Since microbes or diseases appearing in the same cluster are more likely to behave similarly, according to the above conclusion, we construct the graph matrices *S*^m^^*^ and *S*^d^^*^ in terms of microbe space and disease space respectively on the basis of the *p* nearest neighbors and corresponding clustering information. Here, we use the ClusterONE method (Nepusz et al., 2012) to construct the graph *S*^m^^*^from microbe space, in which the weight matrix *X*^*m*^ is generated based on the microbe similarity matrix *S*^*m*^ as follows:

(19) $$\begin{array}{l} X_{ij}^{m}=\{\begin{array}{l} 1\;i\in N\text{(}m_{j}\text{) ​​}\&\text{​​ }j\in N\text{(}m_{i}\text{),}m_{j}\in C\\ 0\;i\notin N\text{(}m_{j}\text{) ​​}\&\text{​​ }j\notin N\text{(}m_{i}\text{),}m_{j}\notin C\\ 0.5\;otherwise \end{array} \end{array}$$

where *N*(*m*_*i*_) and *N*(*m*_*j*_) are the sets of *p* nearest neighbors of *m*_*i*_ and *m*_*j*_, respectively. *C* denotes to any one of the clusters obtained by ClusterONE method and we define the graph matrix *S*^m^^*^ for microbes as follows:

(20) $$\begin{array}{l} \forall i,j\;S_{ij}^{m^{*}}=X_{ij}^{m}S_{ij}^{m} \end{array}$$

In a similar way as the computation of *S*^m*^, we calculate the graph matrix *S*^d^* according to the disease similarity matrix *S*^*d*^.

Here, we defined Φ = [ φ_*ik*_ ] and Ψ = [ ψ_*jk*_ ] as the Lagrange multipliers for the constrains *w*_*ik*_ ≥ 0 and *h*_*jk*_ ≥ 0, respectively. In this work, we first convert the optimization problem in Equation (15) to an unconstraint problem, then minimize this problem by utilizing the corresponding Lagrange function *L*_*f*_ as follows:

(21) $$\begin{array}{ll} L_{f}=Tr\left(YY^{T}\right)-2Tr\left(YHW^{T}\right)+Tr\left(WH^{T}HW^{T}\right)+\lambda_{l}Tr\left(WW^{T}\right)\\ + & \lambda_{l}Tr\left(HH^{T}\right)+\lambda_{m}Tr\left(W^{T}L_{m}W\right)+\lambda_{d}Tr\left(H^{T}L_{d}H\right)\\ + & Tr\left(\Phi W^{T}\right)+Tr\left(\text{Ψ}H^{T}\right) \end{array}$$

To solve the above problem, we first calculate the partial derivatives with respect to *W* and *H* as follows:

(22) $$\begin{array}{l} \frac{\partial L_{f}}{\partial W}=-2YH+2WH^{T}H+2\lambda_{l}W+2\lambda_{m}L_{m}W+\Phi \end{array}$$

(23) $$\begin{array}{l} \frac{\partial L_{f}}{\partial H}=-2Y^{T}W+2HW^{T}W+2\lambda_{l}H+2\lambda_{d}L_{d}H+\text{Ψ} \end{array}$$

After using the Karush-Kuhn-Tucker (KKT) conditions of φ_*ik*_*w*_*ik*_ = 0 and ψ_*jk*_*h*_*jk*_ = 0 (Facchinei et al., 2014), we can obtain the equations for *w*_*ik*_ and *h*_*jk*_ as follows:

(24) $$\begin{array}{ll} - & {\left(YH\right)}_{ik}w_{ik}+{\left(WH^{T}H\right)}_{ik}w_{ik}+{\left(\lambda_{l}W\right)}_{ik}w_{ik}\\ + & {\left[\lambda_{m}\left(D_{m}-S^{m^{*}}\right)W\right]}_{ik}w_{ik}=0 \end{array}$$

(25) $$\begin{array}{ll} - & {\left(Y^{T}W\right)}_{jk}h_{jk}+{\left(HW^{T}W\right)}_{jk}h_{jk}+{\left(\lambda_{l}H\right)}_{jk}h_{jk}\\ + & {\left[\lambda_{d}\left(D_{d}-S^{d^{*}}\right)H\right]}_{jk}h_{jk}=0 \end{array}$$

Finally, on the basis of the above two equations, we can get the updating rules for *w*_*ik*_ and *h*_*jk*_ as follows:

(26) $$\begin{array}{l} w_{ik}\leftarrow w_{ik}\frac{{\left(YH+\lambda_{m}S^{m^{*}}W\right)}_{ik}}{{\left(WH^{T}H+\lambda_{l}W+\lambda_{m}D_{m}W\right)}_{ik}} \end{array}$$

(27) $$\begin{array}{l} h_{jk}\leftarrow h_{jk}\frac{{\left(Y^{T}W+\lambda_{d}S^{d^{*}}H\right)}_{jk}}{{\left(HW^{T}W+\lambda_{l}H+\lambda_{d}D_{d}H\right)}_{jk}} \end{array}$$

**Figure 2:**
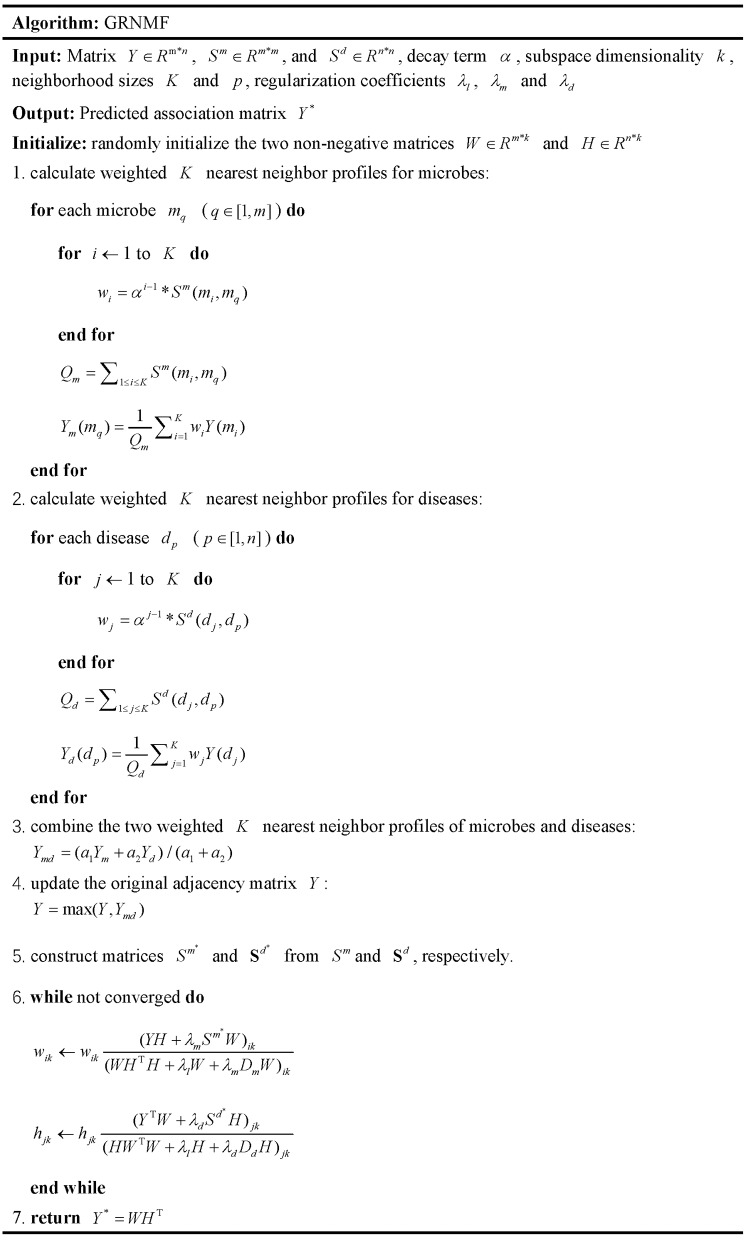
The pseudocode of the whole GRNMF algorithm.

Based on the above two updating formulas, we can obtain the final two non-negative matrices *W* and *H* until convergence. Subsequently, we calculate the score matrix Y^*^ for microbe-disease pairs by utilizing Y^*^ = *WH*^T^, in which the higher score of a microbe-disease pair indicates that the microbe is more likely to be associated with the corresponding disease. In addition, for better understanding, we provided the pseudocode of the whole GRNMF algorithm (See Figure 2).

## Results

### Performance evaluation

Cross validation, a widely used assessment method, was introduced to evaluate the prediction performance of GRNMFHMDA. In this study, we utilized two types of cross validations, namely, global LOOCV and local LOOCV. For the global LOOCV, each of the known microbe-disease associations was in turn considered to be the test sample while the remaining known associations were treated as the training samples. Besides, all of the unknown microbe-disease pairs were regarded as the candidate samples which would be used in the ranking process. After implementing GRNMFHMDA, we ranked each test sample with all candidate samples according to their predicted scores. As for local LOOCV, the difference is that the test sample was only ranked with the candidate samples involving the investigated disease.

**Figure 3:**
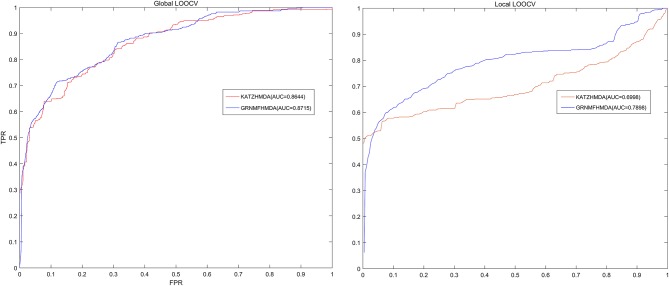
The comparison of prediction performance between GRNMFHMDA and the classical model of KATZHMDA both in global LOOCV and local LOOCV. As a result, GRNMFHMDA achieved AUCs of 0.8715 and 0.7898 in the global and local LOOCV, which exceed the first computational model of KATZHMDA in the field of microbe-disease association prediction.

In each cross validation process, we would consider that the test sample was successfully predicted if the ranking of the test sample was higher than the given threshold. Further, based on the ranks of all test samples, we drew a receiver operating characteristic (ROC) curve through calculating the ratio between true positive rate (TPR, sensitivity) and false positive rate (FPR, 1-specificity) under different thresholds both for global LOOCV and local LOOCV. Sensitivity meant the ratio between the number of test samples ranking higher than the given threshold and the number of positive samples (known microbe-disease associations), while 1-specificity denoted the percentage of the number of negative microbe-disease pairs whose ranks were lower than the given threshold. Moreover, area under the ROC curve (AUC) was calculated to make quantitative evaluation for our model's prediction performance. The model would be considered to be able to perfectly predict all associations if the value of AUC equaled to 1, while the model was only supposed to be able to make random prediction if the value of AUC equaled to 0.5. As a result, GRNMFHMDA obtained AUCs of 0.8715 and 0.7898 in global LOOCV and local LOOCV, respectively. Furthermore, the prediction performance of our model outperformed the KATZHMDA both in global LOOCV (0.8644) and local LOOCV (0.6998), which proved the superior accuracy and reliability of our model in predicting microbe-disease associations (See Figure 3).

### Case study

Here, we put forward two types of case studies on three different common human diseases with the purpose of further assessing the prediction performance of GRNMFHMDA. On the basis of the known microbe-disease associations in HMDAD, we implemented GRNMFHMDA to predict disease-related microbes and then validated the top 10 predicted microbes by HMDAD or recent literatures.

Asthma, a common long-term inflammatory disease of the airways of the lungs, often starts during childhood and its average number of deaths and death rates (per 100,000 people) respectively reached to 38 and 0.1 in 2016 in the World Health Organization (WHO) European region among 10–14 years old children (Kyu et al., 2018). Here, under the GRNMFHMDA framework, asthma was treated as an investigated disease to explore its potential associated microbes. As a result, 9 out of the top 10 microbes in the prediction list were confirmed to be associated with asthma by experimental literatures (See Table 1). For example, *Lactobacillus casei rhamnosus* Lcr35, a species of *Lactobacillus* (1st in the prediction list), was found to be able to attenuate airway inflammation and hyperreactivity in a mouse model of asthma through oral treatment before sensitization (Yu et al., 2010). Besides, Ding et al. (2018) discovered that exosomes derived by *Pseudomonas* (2nd in the prediction list) *aeruginosa* could induce protection against allergic sensitization in asthma mice. Another example is that there is a distinct alteration of the sputum microbiota with a greater prominence of *Firmicutes* (4th in the prediction list) in severe asthma (Zhang et al., 2016).

**Table 1:** Prediction list of the top 10 potential asthma-related microbes based on the known associations in HMDAD database and the corresponding validation evidences (experimental literatures in PubMed) for these associations.

**Rank**	**Microbe**	**Evidence**
1	Lactobacillus	PMID: 20592920
2	Pseudomonas	PMID: 29795208
3	Burkholderia	unconfirmed
4	Firmicutes	PMID: 27078029
5	Actinobacteria	PMID: 23265859
6	Clostridium coccoides	PMID: 21477358
7	Streptococcus	PMID: 17928596
8	Clostridia	PMID: 22047069
9	Lachnospiraceae	PMID: 26512904
10	Fusobacterium	PMID: 24024497

Obesity, a medical condition in which accumulated excess body fat reaches a certain level that may have a negative effect on health, is a leading preventable cause of death worldwide (Reinier and Chugh, 2015). In recent years, plenty of studies have shown certain associations between obesity and microbes that helps a lot to the prevention and treatment of obesity. For instance, many researchers have demonstrated that *methanogens* play a specific role in weight gain and the development of obesity in human subjects (Armougom et al., 2009; Krajmalnik-Brown et al., 2012). Not only that, many studies have now been conducted into the potential of probiotics to ameliorate obesity and diabetes (Delzenne et al., 2011; Peterson et al., 2015). Therefore, taking obesity as another investigated disease in the first type of case study, we found that 9 out of the top 10 predicted obesity-related microbes were confirmed by experimental literatures (See Table 2). For the phylum *Proteobacteria* (1st in the prediction list) which belongs to gram-negative bacteria, the existing study already discovered that it was abundant in the obese group compared with lean group (Park et al., 2015). Besides, as a species of *Clostridia* (2nd in the prediction list), the presence of *Clostridium ramosum* in simplified human intestinal (SIHUMI) enhanced diet-induced obesity according to the experiment data of Woting et al. (2014). Moreover, *Bacillus*, a genus of *Clostridia* (3rd in the prediction list), was found to have outgrown dramatically in the obesity group by Gao et al. (2018).

**Table 2:** Prediction list of the top 10 potential obesity-related microbes based on the known associations in HMDAD database and the corresponding validation evidences (experimental literatures in PubMed) for these associations.

**Rank**	**Microbe**	**Evidence**
1	Proteobacteria	PMID: 25407880
2	Clostridia	PMID: 25271283
3	Bacilli	PMID: 29280312
4	Faecalibacterium prausnitzii	PMID: 19849869
5	Clostridium	PMID: 23645850
6	Betaproteobacteria	PMID: 29312822
7	Clostridium coccoides	PMID: 23147032
8	Lactobacillus	PMID: 23056479
9	Fusobacterium nucleatum	unconfirmed
10	Prevotella	PMID: 21695273

More than that, in order to facilitate future researchers to study the disease-related microbes that they are interested in, based on the known associations in HMDAD, we provided the whole prediction list including all pairs between 292 microbes and 39 diseases as well as their predicted association scores (See Supplementary Table 1).

In addition, to prove the predictive applicability of our model on new diseases without known associated microbes, we carried out another case study on a disease via removing all its known associations in HMDAD. In this way, the prediction process of seeking the investigated disease-related microbes can only depend on the information of other known microbe-disease associations (training samples) and the relevant similarity measures. What needs to be emphasized is that only candidate samples (all microbe-disease pairs including the investigated disease) were ranked and then verified in HMDAD. Hence, there was no overlap between training samples and prediction list. In other words, the verification of predicted associations was independent of HMDAD. Type 1 diabetes, a form of diabetes mellitus, is believed to involve a combination of genetic and environmental factors such as dietary agents (Serena et al., 2015), viral infections (Rewers and Ludvigsson, 2016) and gut microbiota (Bibbò et al., 2017). Especially in gut microbiota, the previous study confirmed that the genus *Bacteroides* is the largest representative of type 1 diabetes-associated dysbiosis that can be modulated by diet (Mejjía-León and Barca, 2015). Thus, considering the significance of studying type 1 diabetes-related microbes, we took type 1 diabetes as the investigated disease to predict its potential associated microbes under the framework of the second type of case study. After implementing GRNMFHMDA, we obtained the ranks of type 1 diabetes' candidate microbes in terms of their association scores (See Table 3). As a result, 8 out of the top 10 predictions were confirmed by HMDAD or recent literatures. For example, Giongo et al. (2011) demonstrated that the *Clostridia* (1st in the prediction list) sequences increased in control samples (samples of general population) as the abundance of *Clostridia* decreased overtime in the case samples (samples of patients with type 1 diabetes). Moreover, at the phylum level and at *p*-values <0.001, *Proteobacteria* (2nd in the prediction list) was found to be higher in case samples than that in control samples (Brown et al., 2011). Another example is that *Lactobacillus strains* (a species of *Lactobacillus* ranking 4th in the prediction list) was found to be able to induce specific changes in the immune system of non-obese diabetic (NOD) mice that can increase or decrease diabetes (Brown et al., 2011).

**Table 3:** Prediction list of the top 10 potential type 1 diabetes-related microbes via removing all the known type 1 diabetes-microbe associations in HMDAD database.

**Rank**	**Microbe**	**Evidence**
1	Clostridia	confirmed by HMDAD
2	Proteobacteria	confirmed by HMDAD
3	Clostridium coccoides	unconfirmed
4	Lactobacillus	confirmed by HMDAD
5	Bacteroidetes	confirmed by HMDAD
6	Firmicutes	confirmed by HMDAD
7	Faecalibacterium prausnitzii	PMID: 23934614
8	Clostridium	PMID: 23433344
9	Betaproteobacteria	unconfirmed
10	Bacilli	PMID: 24930037

*The validation evidences denote to whether the predicted associations were confirmed by the HMDAD database or experimental literatures in PubMed*.

According to the results presented, GRNMFHMDA consistently achieved an excellent predictive performance in the two types of case studies. With the continuous experimental research on microbe-disease associations, we expect that more and more microbes in the prediction lists generated by our model would be verified in the future.

## Discussion

In this article, we proposed a novel prediction model of GRNMFHMDA based on the known microbe-disease associations in HMDAD, Gaussian interaction profile kernel similarity of microbes and diseases and symptom-based disease similarity. To eliminate the possible problem caused by unknown microbe-disease pairs that may affect our final prediction performance, we first implemented a preprocessing step to establish new interaction profiles both for microbes and diseases. Then, after introducing Tikhonov (*L*_2_) and graph Laplacian regularization under the standard NMF framework, we finally obtained reliable and satisfactory prediction performance both in LOOCV and case studies. Therefore, we can conclude that our prediction model is able to play critical role in revealing the associations between microbes and diseases, thus improving the prevention, diagnosis and treatment of many complex human diseases in the future.

Here, the reason why GRNMFHMDA performed well in microbe-disease association prediction lies in the following facts. Firstly, in the study of Wang et al. (2015), to model cancer hallmark traits and networks, nodes and links in the network were weighted, and certain scoring functions were developed to represent gene regulatory logics/strengths on networks. Inspired by that, based on the data extracted from the acknowledged databases, we implemented proper and effective measurements to quantify microbe-disease association network, microbe similarity network and disease similarity network, which guaranteed the reliable prediction performance of our model. Secondly, before implementing GRNMF, we constructed new interaction profiles both for microbes and diseases to further assign those unknown microbe-disease pairs with associated likelihood score, which also improved our model's performance in some degree. Thirdly, different from the standard NMF, we introduced Tikhonov (*L*_2_) and graph Laplacian regularization that ensured the final two non-negative matrices smoothness and guaranteed a part-based representation via fully exploiting the data geometric structure, respectively.

Nevertheless, here are also some limitations restricting the accuracy of our model that need to be overcome in future studies. Initially, the types of similarities for microbes and diseases are not enough yet and we believe that our model would be significantly improved with more biological data and similarity measurements being taken into consideration. Successful advance in association prediction research in various fields of computational biology would also accelerate the development of effective models for microbe-disease association prediction (Chen and Yan, 2013; Chen et al., 2016, 2017b, 2018a,b,c; Chen and Huang, 2017; You et al., 2017). Secondly, as shown in the research of Hao et al. (2018), three representative genome-scale cellular networks, genome-scale metabolic network (GMN), transcriptional regulatory network (TRN), and signal transduction network (STN), were found to be able to become a necessary tool in the systematic analysis of microbes through network integration. Therefore, whether there are similar molecular networks between two microbes is well worth studying in constructing our prediction model. Thirdly, the selection of the optimal parameters is still worth studying. Finally, GRNMFHMDA would inevitably cause bias to diseases that have more known associated microbes and vice versa. Hence, we would come up with optimization strategies to deal with those limitations in our next work.

## Author contributions

B-SH conceived the project, developed the prediction method, analyzed the result, and revised the paper. L-HP designed the experiments, implemented the experiments, analyzed the result, and wrote the paper. ZL analyzed the result and revised the paper. All authors read and approved the final manuscript.

### Conflict of interest statement

The authors declare that the research was conducted in the absence of any commercial or financial relationships that could be construed as a potential conflict of interest.
